# Advances in the Genetic Classification of ALS

**DOI:** 10.1097/WCO.0000000000000986

**Published:** 2021-10-01

**Authors:** Johnathan Cooper-Knock, Calum Harvey, Sai Zhang, Tobias Moll, Ilia Sarah Timpanaro, Kevin P. Kenna, Alfredo Iacoangeli, Jan H Veldink

**Affiliations:** 1 Sheffield Institute for Translational Neuroscience (SITraN), University of Sheffield, Sheffield, UK; 2 Department of Genetics, Stanford University School of Medicine, Stanford, CA, USA; 3 Center for Genomics and Personalized Medicine, Stanford University School of Medicine, Stanford, CA, USA; 4 Department of Neurology, UMC Utrecht Brain Center, University Medical Center Utrecht, Utrecht University, Utrecht, The Netherland; 5 Maurice Wohl Clinical Neuroscience Institute, Department of Basic and Clinical Neuroscience, Institute of Psychiatry, Psychology & Neuroscience, King’s College London, London, UK; 6 Department of Biostatistics and Health Informatics, Institute of Psychiatry, Psychology and Neuroscience, King’s College London, London, UK; 7 National Institute for Health Research Biomedical Research Centre and Dementia Unit, South London and Maudsley NHS Foundation Trust and King’s College London, London, UK

**Keywords:** Amyotrophic lateral sclerosis, genetics, personalised medicine

## Abstract

**Purpose of review:**

Amyotrophic lateral sclerosis (ALS) is an archetypal complex disease where disease risk and severity are, for the majority of patients, the product of interaction between multiple genetic and environmental factors. We are in a period of unprecedented discovery with new large-scale genome-wide association study (GWAS) and accelerating discovery of risk genes. However, much of the observed heritability of ALS is undiscovered and we are not yet approaching elucidation of the total genetic architecture which will be necessary for comprehensive disease subclassification.

**Recent findings:**

We summarise recent developments and discuss the future. New machine learning models will help to address nonlinear genetic interactions. Statistical power for genetic discovery may be boosted by reducing the search-space using cell-specific epigenetic profiles and expanding our scope to include genetically correlated phenotypes. Structural variation, somatic heterogeneity and consideration of environmental modifiers represent significant challenges which will require integration of multiple technologies and a multidisciplinary approach including clinicians, geneticists and pathologists.

**Summary:**

The move away from fully penetrant Mendelian risk genes necessitates new experimental designs and new standards for validation. The challenges are significant but the potential reward for successful disease subclassification is large-scale and effective personalized medicine.

## Introduction

Amyotrophic lateral sclerosis (ALS) is a rapidly progressive and universally fatal late age of onset neurodegenerative disease involving loss of motor neurons. ALS is relatively common with a lifetime risk of ~1/400 (1). ALS is also an archetypal complex disease where 10% of patients suffer monogenic disease but the majority of disease, known as sporadic, is determined by an interaction of multiple environmental and genetic risk factors. More than 30 ALS genes have been previously identified; in monogenic disease the most frequent mutations are found within *C9ORF72*, *SOD1*, *TARDBP* and *FUS*. Changes in known ALS genes are found in ~21% of ALS patients, and the presence of more than one variant is associated with lower age of onset (2). However, there is still substantial missing heritability which is not explained by known ALS genes. It seems likely that the majority of ALS patients carry multiple risk variants which have not yet been identified. Our ability to profile and subdivide sporadic ALS is likely to determine the future of personalised medicine interventions for the majority of ALS patients.

**Figure 1:**
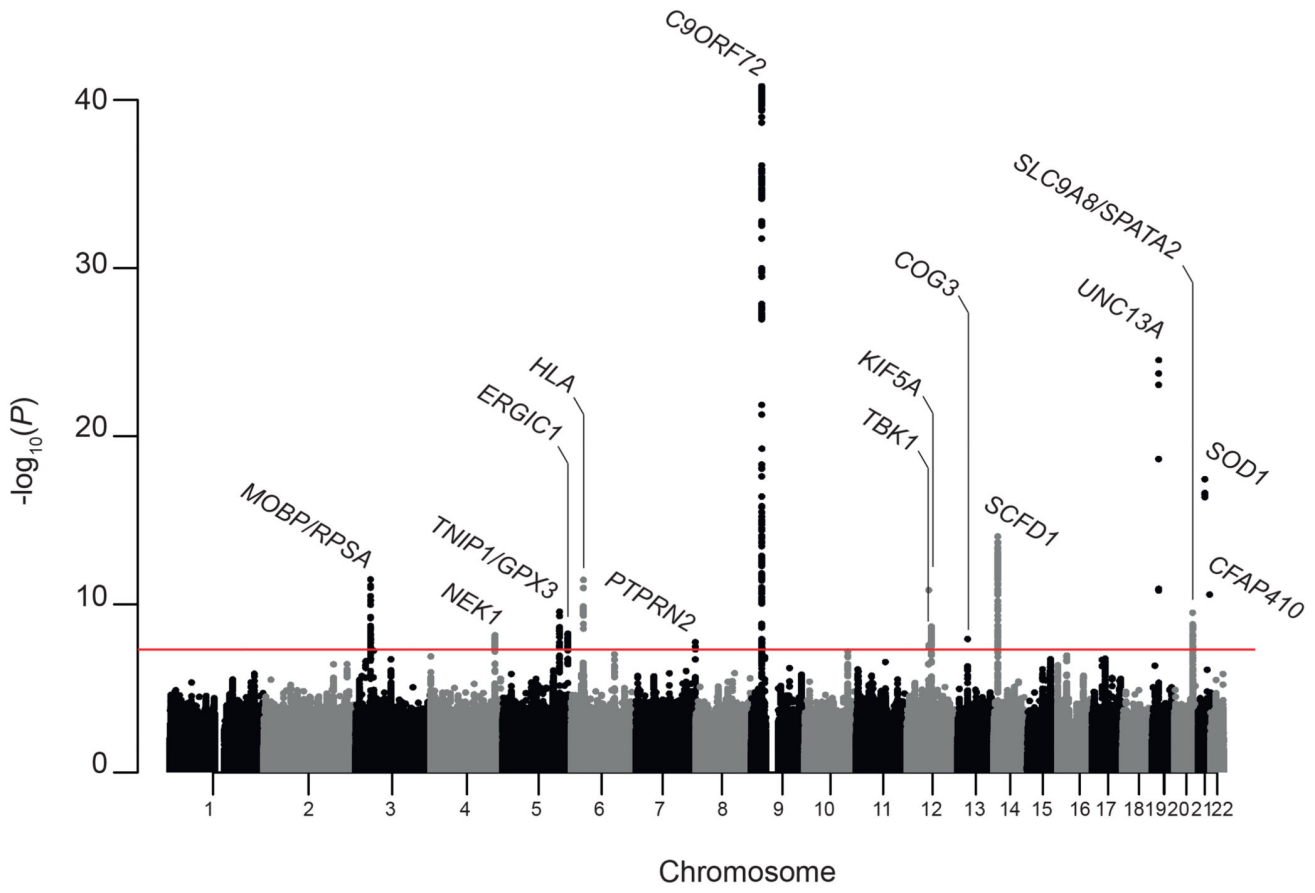
Manhattan plot for new ALS genome-wide association study (GWAS) including cross-ancestry meta-analysis. Red line represents the p-value threshold for genome-wide significance based on Bonferroni multiple testing correction (p=5e-08). Genes linked to loci by prioritization analysis are labelled.

The world is in the midst of the global COVID-19 pandemic which has significantly impeded research progress. Despite this there has been significant progress in the field of ALS genetics. A new ALS genome-wide association study (GWAS) has recently been reported (3). The large sample size of this new study is a significant step forwards and the authors have reported 8 new genome-wide significant loci (Figure 1). In addition several new ALS risk genes have been reported in recent years including *ATXN1 (4)*, *CAV1 (5), SPTLC1 (6), ACSL5 (7,8)*, *DNAJC7* (9), ANXA11 (10) and *GLT8D1* (11). There is a move away from fully penetrant genetic changes, and a move towards identification of genetic risk factors which are probably pathogenic only in conjunction with other genetic and environmental risk factors. Indeed the majority of monogenic disease has now been explained (12) and remaining undiscovered genetic changes with complete penetrance are likely to affect relatively small numbers of pedigrees.

Broad sense heritability for sporadic ALS is ~50% (13,14). This contrasts sharply with the estimates of SNP-based heritability from GWAS which are as low as 3.5% (3). More research is needed to identify the source of this missing heritability. GWAS estimates of heritability typically utilise linear methods which likely underestimate the effect of nonlinear interactions, which may be important in a complex biological system. In contrast, broad sense heritability is often calculated using relatives where nonlinear effects can be profiled more accurately. Using a hypothetical model we have previously shown that linear methods can dramatically fail to identify nonlinear heritability (15). Alternatively, modelling by others has suggested that the impact of nonlinear effects may be negligible (16). Currently, genetic association studies are still dominated by linear methods which, like the heritability estimates, may be underpowered to identify certain genetic risk factors. New, nonlinear methodology is required to answer this question definitively.

A limiting factor in the discovery of ALS genetic risk loci is poor statistical power despite increasing sample sizes. This is an important reason why <10% of SNP-based heritability has been assigned to a specific locus. We have recently demonstrated that statistical power can be improved by reducing the search space to areas of the genome which are functional in a cell type of interest, such as motor neurons (17). Recent advances in single cell profiling of relevant CNS tissues promise much in this regard (18,19). Moreover, integration of other biological data such as protein-protein interaction data and known gene functions can further aid gene prioritization (20,21)

**Figure 2:**
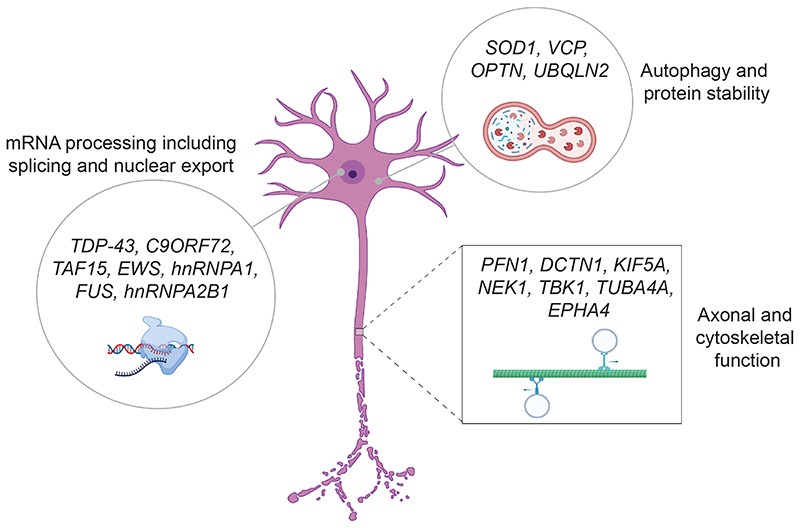
Existing ALS risk genes converge within biological pathways including mRNA processing, autophagy and axonal function. Figure indicates example risk genes and relevant subcellular localisation.

The challenge of genetic classification is to group patients according to management strategy. It is noteworthy that risk genes increasingly converge on certain biological pathways (Figure 2) which may represent distinct therapeutic targets. It has been known for some time that approximately 98% of ALS patients share TDP-43 mislocalization (22) and new research linking this change to molecular and clinical phenotypes suggests that this may represent a final common pathway of ALS amenable to therapeutic intervention (23–25). Cell and animal models are important for drug development, but are usually produced via genetic manipulation and therefore rely on accurate genetic classification. Mistakes in this area can be significant, for example therapies developed for the SOD1-mouse model of ALS have largely failed to translate because SOD1-ALS is not representative of the majority of ALS patients having a distinct molecular basis and neuropathology (26).

## Impact of new GWAS

The new GWAS from van Rheenen et al (3) includes a meta-analysis of 117 cohorts, 29,612 ALS patients and 122,656 controls, which represents a significant step change in the number of cases compared to previous studies (27,28). The study has reported 15 genome-wide significant loci (Figure 1) of which 8 have previously been identified but 7 are new. The study also included whole genome sequencing of 6,538 ALS patients and 2,415 controls which aided comparison of rare and common variant signals. With imputation (29), the GWAS included variants with minor allele frequency (MAF) >0.1% whereas whole genome sequencing profiles all variants. In brief, all identified loci fall within four categories: 1) Rare variants within coding sequence which likely to be directly causal, e.g. within *SOD1*, *KIF5A* and *CFAP410*; 2) Single nucleotide polymorphisms (SNPs) that are in linkage with a pathogenic repeat expansion such as the G4C2-repeat expansion within *C9ORF72*; 3) Loci that consist of both common regulatory variants and rare coding variants that are distinct (not in significant linkage) but have overlapping functional consequences, e.g. variants associated with *NEK1* and *TBK1; 4)* Lastly, remaining loci are those where there is no direct link to a causal gene through coding variants or repeat expansions; these are likely to have relatively subtle effects mediated through as yet unknown changes in gene and protein expression.

Both regulatory and coding variants within the *NEK1* and *TBK1* loci are thought to cause loss of function (LoF) of the target gene in ALS patients. Similarly we have profiled ALS-associated regulatory variation and identified other non-GWAS hits where there is a convergence of common and rare variant signal in a LoF mechanism (5,17). Moreover, a similar phenomenon has been reported in other disease areas (30). Regulatory variants are thought to have lower effect size and more redundancy (31) whereas coding variants typically show higher penetrance. In reality this is likely to be more of a spectrum where population frequencies may be a measure of selection pressure and indirectly effect size.

The new GWAS also took advantage of growing genetic profiles of other diseases to measure genetic correlation between neurodegenerative diseases including progressive supranuclear palsy (PSP), Alzherimer’s disease (AD), Parkinson’s disease (PD) and frontotemporal dementia (FTD). This is an area of increasing understanding but it is clear that ALS-associated risk variants can be associated with multiple phenotypes, most notably FTD. This has been observed previously in the context of populations (32) and pedigrees (33,34). Shared genetic risk amongst different phenotypes may also go some way to explain why ALS is strongly associated with rare variants (28) suggesting a role for selection pressure, despite a late age of onset that should have a minimal effect on reproductive fitness. It is potentially significant that ALS-associated mutations have been associated with molecular mechanisms underlying fertilisation (35).

To date genetic association studies have largely relied on short sequencing reads of up to 150bp. Accurate assessment of SNPs is achieved, but there is an inherent uncertainty in assessment of variants which are longer than the basic read length. This is compounded by a positive bias towards sequence of intermediate GC-content (36) which can reduce coverage of repetitive genomic regions. A key example is the failure for many years to identify the intronic G4C2-repeat expansion within *C9ORF72* which is the most common genetic cause of ALS and FTD (37,38). A number of other repeat-expansions have been associated with ALS including short-tandem repeats (STR) (4,39) and variable-number tandem repeats (40). The new GWAS (3) includes a genome-wide screen for ALS-associated STR using short read sequencing data. This is achieved by taking advantage of previously annotated STRs (41) and identification of anchored read pairs containing repetitive sequence and adjacent sequence which can be confidently mapped to the genome (42). Expansion of a STR downstream of NEK1, a known ALS gene (43), was thus significantly associated with ALS risk.

## Genetic classification of a polygenic disease

The promise of genetic classification is nothing short of personalised medicine. For monogenic disease this is already becoming a reality (44). However, a prerequisite for widely applicable subclassification is an approximation of the total genetic architecture of ALS. Current efforts to capture genetic architecture are dominated by polygenic risk scores (PRS) developed from GWAS data (45) but this is a linear method and success has been limited (46). A recent effort to produce a PRS for ALS (47) reported limited ability to provide individualised predictions with a maximum AUC of only 0.57. Advances in machine learning have led to new tools such as deep learning, which are the state of the art in many classification problems (48) and hold promise for analysis of complex biological systems (49). Machine learning approaches offer an opportunity to integrate multiple data-types; a model based on a graph representation of protein-protein interactions, known gene functions and gene-disease associations has been proposed for the prediction of novel ALS genes (20). A retrospective validation study of predictions made using this model in early 2019, revealed that those predictions were enriched (p=0.012) for subsequently discovered ALS genes (21).

It is suggested that ALS is a multistep process involving both genetic and environmental factors (50,51). As a result it may be impossible to effectively classify ALS based on genetic variation in isolation. Progress has been made to identify environmental modifiers of ALS risk using Mendelian randomisation (MR), including strenuous exercise (52) and blood lipids (3,53). MR relies on measured genetic liability to a particular environmental exposure and therefore it is amenable to study of the gene-environment interaction (54). However, efforts to perform this at scale will likely require either clear hypotheses, or large sample sizes. The gut microbiome is an effective integrator of environmental effects (55) and therefore microbiome profiling alongside host genetics may be an effective method of measuring gene-environment interactions. By matching multiple profiles within the same patients, including for example metabolome and microbiome, it may be possible to boost power to identify new genetic risk while simultaneously determining the target group. Such multi-omics data is increasingly available from consortia such as AnswerALS (https://www.answerals.org/).

In a fascinating development, the new ALS GWAS (3) has shown that severity and risk of ALS are genetically independent (3). The suggestion is that genetic classification, and therefore identification of effective therapeutic targets, may require integration of separate risk and severity signals.

## ALS risk genes converge in pathways

The new ALS GWAS (3) took advantage of a new RNA-seq dataset including cortical tissues from 31,684 samples known as Metabrain (56). Using brain specific co-expression networks the authors localised ALS genetic risk to Gene Ontology terms related to vesicle mediated transport and autophagy. Vesicle mediated transport is involved in synaptic neurotransmission but also in vesicle fusion, which is an essential component of autophagy (57). Our study of the genetic architecture of ALS in the context of motor neuron function highlighted function within the distal axon (17) and demonstrated that axonal dysfunction associated with LoF of *KANK1*, a putative new ALS gene, may be upstream of TDP-43 mislocalization.

Various databases have complied lists of ALS risk genes and variants including ALSoD (https://alsod.ac.uk/), GCEP (https://clinicalgenome.org/tools/educational-resources/gene-disease-validity-topics/gcep-presentations/) and ClinVar (https://www.ncbi.nlm.nih.gov/clinvar/?term=amyotrophic+lateral+sclerosis). Overall there is an observed convergence of risk genes within a relatively small number of pathways (58) including cytoskeletal dynamics, protein homeostasis including autophagy, RNA processing and immune cell function (Figure 2). The observed convergence may be subject to some publication bias which is difficult to combat. However, overall there is good evidence that these pathways should be subject to an intense search for effective therapeutic targets, and future genetic subclassification may be focused on these pathways which have already been identified. However, if severity and risk of ALS are genetically independent then perhaps the field needs to shift efforts to discovery of biological pathways important for disease outcome measures such as age at onset, survival and cognitive impairment.

In the past six months the ALS field is evaluating an evolving story which comes close to a universal pathogenic mechanism. TDP-43 is said to be the central protein for ALS because mislocalization of nuclear TDP-43 within motor neurons and the formation of cytoplasmic neuronal TDP-43-positive inclusions links >98% of ALS patients (22) and predicts the severity of neuronal loss (59). TDP-43 has long been shown to regulate RNA splicing but a specific role in control of splicing of cryptic exons was only discovered in 2015 (60). It has been recently shown that TDP-43 controls the expression of cryptic exons within *UNC13A* mRNA. *UNC13A* is the subject of known ALS GWAS locus where ALS-associated SNPs, are linked to risk of disease (61) and patient survival (25,62). When TDP-43 is lost from the nucleus then cryptic exons are included within the mature *UNC13A* mRNA which are otherwise excluded; in some cases this leads to nonsense mediated decay (NMD) and loss of protein function. Crucially, ALS-associated GWAS SNPs within *UNC13A* exacerbate the effect of TDP-43 dysfunction leading to LoF of UNC13A protein (23,24) suggesting that this mechanism is upstream in disease pathogenesis. Going forwards, cryptic exon inclusion may become a unifying theme in ALS pathology; new discoveries may need to be evaluated for an interaction with TDP-43 mislocalization and/or UNC13A. For example, it has been shown that TDP-43 normally suppresses a cryptic polyadenylation site within the axonal protein STMN2; loss of nuclear TDP-43 leads to NMD of the *STMN2* mRNA and near-total knock-down of the protein (63,64). LoF of STMN2 has been associated with motor neuron toxicity (65), genetic variants within *STMN2* have been associated with ALS severity (66) and the interaction between TDP-43 and *STMN2* mRNA is the focus of active translational research.

## Future Directions for discovery of ALS genetic risk

We have already stated that the prospect for discovery of new monogenic changes in ALS is now limited to what are likely very rare mutations with undetermined relevance for the majority of patients. We have argued that new approaches are required to address complex inheritance patterns in sporadic ALS including nonlinear machine learning models, integration of epigenetic profiling in vulnerable cell types, recognition of associated pleiotropic phenotypes (particularly FTD) and correlation with measures of environmental risk factors to draw out gene-environment interactions. The case-control design that informs many GWAS and even rare variant studies, such as Project MinE (67), is effective but increasing sample sizes are unlikely to be sufficient alone to delineate a total genetic architecture of ALS, given the inherent lack of phenotypic detail. On the other hand, classical, family based studies will also be of limited value, given the large degree of incomplete penetrance and partly recognized pleiotropy.

Fundamentally genetic risk is inherited and therefore in complex diseases, family members carry higher than background genetic risk even if they are unlikely to develop disease (68). As a result, exclusion of unaffected family members is likely to lead to a loss of substantial statistical power. To take advantage of this we purpose a ‘super pedigree’ approach utilising non-Mendelian pedigrees with variable penetrance and pleiotropy. The focus would be on identification of drivers of genetic risk of ALS and related disorders. Candidate related disorders are dyslipidemia, body mass index (BMI), cognitive disorders and psychiatric disease.

We have described the recent discovery of several structural variants associated with ALS including STRs and VNTRs. Similar technology should allow more accurate profiling of copy number variants (CNV) a number of which have been associated with rare instances of ALS (69). Interestingly TDP-43 mislocalization has been associated with aberrant expression of LINE1 retrotransposons (70) which are a frequent source of structural variation within the genome. It is likely that a significant number of as yet undiscovered structural variants are associated with ALS. Long-read sequencing technology is leading to advances in this area (71) but cost, throughput and accuracy may prohibit large-scale genome wide long read sequencing studies for some time (72). The near future is likely to be dominated by improvements in imputing structural variants from short read sequencing data, perhaps using models trained using long read sequencing; indeed several exciting new genome builds are being released (73) which will be essential in this area.

Finally, there is a case for somatic mosaicism in ALS. Somatic mosaicism could in theory explain a number of observed phenomena in ALS: 1) The adult onset of the disease which could result from accumulation of mutations within transcriptionally active brain regions (74); 2) The multifocal and heterogeneous onset of disease; 3) The failure to delineate either a total genetic architecture or establish broadly relevant environmental risk factors for ALS (75); 4) The selective involvement of particular neuronal populations, particularly within the motor system. Notably, the most common genetic risk factor for ALS, G4C2-repeat expansion of *C9ORF72*, shows marked somatic mosaicism (76). Technical challenges have limited discovery of other somatic mutations, for example it is difficult to distinguish a rare change affected in a limited number of cells from technical variation arising during sequencing (77). Also, by definition mutations which have occurred in dying cells are hard to capture at post-mortem. Somatic heterogeneity has long been studied in the context of cancer biology and perhaps methodology can be adapted from this field as described in (78) and (79). ALS is characterised by selective vulnerability of specific cell populations within the CNS and so non-CNS tissues may be an effective control for technical variation within an individual. However, many existing ALS-associated mutations are notable for their global expression and it may be that non-genetic factors, or even cell-specific functions (17–19), are a more important determinant of selective vulnerability. Addressing this problem will require a multidisciplinary approach involving clinicians, pathologists and geneticists.

## Importance of validation for incompletely penetrant changes

There is already a case to be made that certain ALS-associated mutations are false positives (80). Whilst a period of evaluation is necessary for any new discovery, the ALS field needs to agree consensus on genetic risk factors if we are ultimately to reach a clinical grade test for disease subclassification.

A common cause of false positives is population variability and it is therefore important that case-control designs include matched control populations but also that biological signals replicate in different populations as has been observed in the most recent GWAS (2) where 8 loci were genome-wide significant in European and Asian ancestry groups (Figure 1).

Experimental validation is a helpful tool, particularly when classifying risk genes by pathway and ultimately therapeutic target, as has already been discussed. However, some models are likely to be more representative than others. The field is currently favouring patient-derived models (81) which can recapitulate key molecular pathologies including TDP-43 mislocalization (82) and astrocyte toxicity towards motor neurons (83). These models also have the advantage that they can be adapted for drug screening but still require robust readouts that are relevant to clinical phenotypes. Biobanks such as AnswerALS are producing iPSC-derived motor neurons from patients at scale alongside other omics and clinical data (https://www.answerals.org/) which will facilitate identification of such readouts.

## Conclusion

In conclusion, we are experiencing a rapid expansion in the genetic profiling of ALS headlined by new GWAS (3), the largest disease-specific whole genome sequencing consortium for any disease in Project MinE (https://www.projectmine.com/) and large multi-omics datasets such as AnswerALS (https://www.answerals.org/). This is leading to unprecedented discovery of risk genes and loci and is moving the field away from monogenic fully penetrant changes towards risk variants with variable penetrance which interact with other genetic and environmental factors. This new era brings new challenges with a need for new methods. Future studies will need to account for reduced penetrance, genetic pleiotropy and nonlinear inheritance. Both structural variation within the genome and somatic heterogeneity are promising areas of research. Lastly, we predict that successful genetic subclassification of ALS will have a dramatic positive influence on therapeutic developments, both by directly targeting mutations with genetic therapy approaches but also by enabling the generation of new, more valid ALS models for drug screening to identify neuroprotective compounds.

## Ket Points

- Rapid progress in ALS genetics has led to the discovery of new loci and new genes but does not completely explain the observed heritability.
- The focus of research has moved away from fully penetrant monogenic variants towards risk variants with incomplete penetrance.
- Specific challenges and opportunities for gene discovery include genetic pleiotropy, gene-environment interactions, structural variation and nonlinear genetic interactions.
- A comprehensive genetic architecture for ALS could facilitate disease subclassification and lead to personalised medicine.
